# Experimental dataset on electrolyte mixtures containing fluoroethylene carbonate and lithium bis(trifluoromethanesulfonyl)imide

**DOI:** 10.1016/j.dib.2019.103703

**Published:** 2019-02-02

**Authors:** Zhengqi Wang, Andreas Hofmann, Thomas Hanemann

**Affiliations:** a Karlsruhe Institute of Technology (KIT), Institute for Applied Materials - Materials and Processes (IAM-WK), Hermann-von-Helmholtz-Platz 1, 76344 Eggenstein-Leopoldshafen, Germany; b University of Freiburg, Department of Microsystems Engineering (IMTEK), Georges-Köhler-Allee 102, 79110 Freiburg, Germany

## Abstract

These data and analyses support the research article “Low-flammable electrolytes with fluoroethylene carbonate based solvent mixtures and lithium bis(trifluoromethanesulfonyl)-imide (LiTFSI) for lithium-ion batteries” [1]. The data and analyses presented here include fitted data for density measurements, temperature dependence of density and specific volume of the mixtures, detailed viscosity measurements and conductivity data, current density plots with respect to anodic aluminum dissolution, half-cell C-rate capability of mixtures with the additives used in research article as well as the SEM images and EDX data of the full-cell with the electrolyte selected and controlled.

## Specifications table

**Table:** 

Subject area	*chemistry*
More specific subject area	*electrochemistry, energy storage*
Type of data	*Table, text file, figure, equation*
How data was acquired	*Density measurements, rheology and conductivity measurements, current-voltage profiles, cell cycling measurements, SEM characteristics, EDX element profile*
Data format	*Raw, analyzed, fitted*
Experimental factors	*Preparation of mixtures of organic carbonates (1:1 M ratio) and LiTFSI inside of an argon filled glovebox*
Experimental features	*The density was obtained by measuring electrolyte mixtures with the device DMA 4500 M from Anton Paar.*
	*Conductivity was measured with a device from RHD instruments by the standard complex impedance method.*
	*The dynamic viscosity was measured using a rotational rheometer (cone/plate geometry, 40/1°, gap of 30 mm, Malvern Gemini HR Nano, Worcestershire, UK) in range of T = (15–80) °C (shear rate of 100 s*^*-1*^*).*
	*The anodic aluminum dissolution was measured in Swagelok-cells. The cells were assembled inside a glove box and were cycled at the range of 2.5–4.3 V (1 mV s*^*-1*^*) at 24–26 °C.*
	*The cell tests were done in coin cell configuration with lithium foil, NMC material, C anode electrode and glass fiber separator. The measurements were done at a cell cycler own-made from the KIT.*
	*The SEM/EDX analyses were done at Zeiss Supra 55 with EDX.*
Data source location	*Eggenstein-Leopoldshafen, Germany*
Data accessibility	*Data is with this article*
Related research article	*Z. Wang, A. Hofmann, Thomas Hanemann;* Low-flammable electrolytes with fluoroethylene carbonate based solvent mixtures and lithium bis(trifluoromethanesulfonyl)imide for Lithium-ion batteries*; Electrochim. Acta; in press.*[1]

## Value of the data

• The temperature-dependent fitting data of the density values are provided.
• The density data are linked to the specific volume of the mixtures.
• Temperature dependent data of viscosity and conductivity give a detailed insight to the electrolyte solvents which is important for battery research.
• The current density diagrams support the microscopy analysis.
• The cell test data demonstrates the usability of the additives VC, LiBOB and LiDFOB.

## 1 Data

In this data manuscript, additional information on the article [1] is provided. The following data are shown in detail: (1) The density data and the parameters by fitting equation of the temperature-dependent density, (2) the temperature-dependent specific volume and the volumetric expansion coefficients of mixtures derived from the density measurements, (3) the temperature-dependent viscosity values, (4) specific and molar conductivity data, (5) time-dependent current density measurements of the anodic aluminum dissolution reactions, (6) half-cell cycle data (Li||NMC and Li|C) of the electrolyte with various selected additives, (7) SEM images and EDX analysis data for the anode materials after full cell cycles. All these data complement the measurements and analyses performed in the manuscript [1].

### 1.1 Temperature dependent density data of all electrolytes

The density data are fitted according to Eq. (1):

(1) ρ(T)=a+b·T

where *a*, *b*, *T* are the density at 0 K, the coefficient of volume expansion (<0, g cm^-3^ K^-1^) and the temperature (K), respectively. The experimental values of density, the fit parameters and the correlation coefficient were shown in Table 1.

**Table 1:** Temperature-dependent density *ρ* data and the adjustable parameters of its equation (ρ=a+b∙T) of electrolyte mixture.

**Sample**	**EM-0**	**EM-1**	**EM-2**	**EM-3**	**EM-4**	**EM-5**	**EM-6**
*ρ*, T = 293.15 K, [g cm^-3^]	1.30	1.59	1.52	1.44	1.39	1.33	1.24
*ρ*, T = 313.15 K, [g cm^-3^]	1.28	1.56	1.49	1.41	1.37	1.31	1.21
*ρ*, T = 333.15 K, [g cm^-3^]	1.26	1.53	1.47	1.39	1.35	1.29	1.19
*ρ*, T = 353.15 K, [g cm^-3^]	1.23	1.50	1.44	1.37	1.32	1.27	1.17
*ρ*, T = 298.15 K, [g cm^-3^]^a^	1.30	1.58	1.51	1.43	1.39	1.33	1.23
*a*, [g cm^-3^]	1.64	1.99	1.88	1.79	1.73	1.63	1.57
*b*, [10^-3^ g cm^-3^ K^-1^]	-1.15	-1.36	-1.25	-1.20	-1.15	-1.00	-1.14
Cor. R^2^, b	1.000	1.000	1.000	1.000	1.000	1.000	1.000

a Value calculated by linear fitting of the temperature-dependent density data;

b Correlation coefficient for linear fitting of the temperature-dependent density data.

### 1.2 Temperature dependence of specific volume of the mixtures

By using the density values (at 25 °C) obtained in Table 1, the specific volume (*V*, cm^3^ g^-1^) of individual component was calculated in the following Table 2. Referred to the preparation, the inverse value of LiTFSI and LiPF_6_ density (1.334 and 1.50 g cm^-3^) are the specific volume *V*_salt_ is 0.750 cm^3^ g^-1^ and 0.667 cm^3^g^-1^, respectively. The density of co-carbonate equimolar mixtures could be obtained using this relationship: $\rho_{12}=\rho_{1}\rho_{2}(M_{1}+M_{2})/(\rho_{1}M_{2}+\rho_{2}M_{1})$, assumed that the volume change after mixing co-carbonates is neglected. Therefore, the specific volume *V*_solvent_ of the co-carbonates were also calculated. The volumes of 1 kg components (salt and carbonates) could be simply summed to be the total volume, *V*_cal_ (cm^3^) = *V*_salt_ + *V*_solvent_, here the volume change after dissolution is not taken into account. In addition, the real total volume *V*_exp_ (cm^3^) of 1 kg mixture could be obtained by using the measured density values.

**Table 2:** The comparison between the experimental specific volume *V*_exp_ data of electrolyte mixtures and the calculated specific volume *V*_cal_ of lithium salts and solvents before mixing.

**Sample**	**EM-0**	**EM-1**	**EM-2**	**EM-3**	**EM-4**	**EM-5**	**EM-6**
*ρ*, T = 298.15 K, [g cm^-3^]^a^	1.30	1.58	1.51	1.43	1.39	1.33	1.23
*V*_exp_, T = 298.15 K, [cm^3^ kg^-1^]	771.37	632.80	662.17	697.82	720.35	752.54	812.74
*V*_salt_, T = 298.15 K, [cm^3^] pro 1 kg salt+solvents	78.11	161.41	161.41	161.41	161.41	161.41	161.41
*V*_solvent_, T = 298.15 K, [cm^3^] pro 1 kg salt+solvents	738.46	521.73	554.52	585.49	606.60	640.56	701.10
*V*_cal_ = *V*_salt_ + *V*_solvent_, T = 298.15 K, [cm^3^] pro 1 kg salt+solvents	816.57	683.14	715.93	746.90	768.01	801.96	862.51
*(V*_cal_*-V*_exp_*)/V*_cal_, [%]	5.54	7.37	7.51	6.57	6.21	6.16	5.77

a Values from Table 1.

The differential of the measured specific volume d*V* at temperature interval d*T* is expressed by following relationship (2):

(2) $$\mathrm{dV}=V_{0}\text{·}\beta \text{·}\mathrm{dT}$$

where *V*_0_, *β*, *T* are the specific volume (cm^3^ g^-1^) at reference temperature (here room temperature), the volumetric thermal expansion coefficient (>0, K^-1^) and the temperature (K), respectively. The volumetric temperature expansion coefficient *β*, as shown in Table 3, reveals the response of volume to the temperature changes and was correlated to the heat on the squeezing/separation of molecules against/from others and to the changes of the coordination structures. For the different structures of carbonates in electrolyte mixtures, an obvious divergence of the volumetric expansion coefficients at a given temperature is observed. The *β* of mixtures with the selected linear carbonates are different, compared to the ones with cyclic carbonates. The solvents EM-5 contains the rigid and conjugated benzyl groups of DBC which shown the thermal expansion stability, leading to a weaker response to the heat. The EM-6 contained the flexible propyl chains –CH_2_CH_2_CH_3_ of DPrC, was more “sensitive” to the temperature increasing and gains larger volume expansion. Additionally, the expansion coefficient *β* shown also a temperature dependent behavior.

**Table 3:** Temperature-dependent specific volume *V* and the average volumetric temperature expansion coefficient *β* of the relationship $\mathrm{dV}=V_{0}∙\beta ∙\mathrm{dT}$ of electrolyte mixtures.

**Sample**	**EM-0**	**EM-1**	**EM-2**	**EM-3**	**EM-4**	**EM-5**	**EM-6**
*V*_0_, 298.15 K, [cm^3^ kg^-1^]^a^	771.37	632.80	662.17	697.82	720.35	752.54	812.74
*V*_1_, 293.15 K, [cm^3^ kg^-1^]	767.95	630.37	659.55	695.49	717.53	750.05	809.62
*V*_2_, 313.15 K, [cm^3^ kg^-1^]	781.82	641.43	670.65	707.40	729.63	761.55	824.85
*V*_3_, 333.15 K, [cm^3^ kg^-1^]	796.17	652.94	682.10	719.65	742.10	773.34	840.70
*V*_4_, 353.15 K, [cm^3^ kg^-1^]	811.06	664.62	693.90	732.29	754.94	785.48	857.23
*β*_12_, 293.15–313.15 K, [10^-4^ K^-1^]	8.99	8.74	8.38	8.53	8.40	7.64	9.37
*β*_23_, 313.15–333.15 K, [10^-4^ K^-1^]	9.30	9.01	8.64	8.78	8.65	7.83	9.75
*β*_34_, 333.15–353.15 K, [10^-4^ K^-1^]	9.65	9.31	8.92	9.05	8.92	8.07	10.17

a The specific volume data were calculated from Table 1.

### 1.3 Viscosity values of electrolyte mixtures EM-0 to EM-6

See Table 4.

**Table 4:** Temperature-dependent viscosity *η* data and the adjustable parameters of its VFT equation ($\eta =\eta_{0}\exp[B/(T-T_{0})])$ of electrolyte mixtures.

**Sample**	**EM-0**	**EM-1**	**EM-2**	**EM-3**	**EM-4**	**EM-5**	**EM-6**
*η*, 288.15 K, [mPa s]	5.0	17.7	15.6	15.7	17.0	62.6	11.3
*η*, 293.15 K, [mPa s]	4.4	14.6	13.1	13.1	14.2	47.6	9.7
*η*, 298.15 K, [mPa s]	3.9	12.3	11.1	11.2	12.0	37.1	8.4
*η*, 303.15 K, [mPa s]	3.5	10.4	9.5	9.7	10.3	29.7	7.4
*η*, 308.15 K, [mPa s]	3.2	9.0	8.4	8.5	9.0	24.3	6.5
*η*, 313.15 K, [mPa s]	3.0	7.8	7.4	7.5	7.9	20.2	5.8
*η*, 323.15 K, [mPa s]	2.6	6.1	5.8	5.9	6.2	14.4	4.7
*η*, 333.15 K, [mPa s]	2.3	4.9	4.7	4.8	5.0	10.9	–
*η*, 343.15 K, [mPa s]	2.0	3.5	3.3	3.5	3.6	7.5	2.9
*η*, 353.15 K, [mPa s]	1.9	2.9	2.7	3.0	2.9	5.9	2.5
*η*_0_, [10^-2^ mPa s]	47.97	19.41	22.9	34.4	29.3	33.1	18.9
*Β*, [K]	205.9	512.0	485.0	392.4	431.5	474.7	528.7
*T*_0_, [K]	200.4	174.7	173.3	185.5	181.9	197.6	158.9
*T*_g_*-T*_*0*_, [K]	5.0	11.5	11.8	-5.7	-4.1	7.3	–
*D = B/T*_0_	1.0	2.9	2.8	2.1	2.4	2.4	3.3
Cor. R^2^, a	0.995	0.999	0.999	0.999	0.999	0.999	0.999
*E*_A,η_, [kJ mol^-1^] b	12.2	23.3	22.3	21.2	22.5	30.3	19.9

a Correlation coefficient for fitting of the temperature-dependent viscosity data using VFT equation.

b The activation energy values *E*_A,η_ were evaluated using the slope (*E*_A,η_*/R*) of the Arrhenius plots. Tentatively treated as a constant between 15 and 80 °C.

### 1.4 Conductivity data of the electrolyte mixtures

See Table 5.

**Table 5:** Temperature-dependent specific conductivity *κ*, molar conductivity *Λ*(Λ=κ∙M/ρ) data and the adjustable parameters of its VFT equation ($\kappa =\kappa_{0}\exp[C/(T-T_{1})]\;\mathrm{and}\;\Lambda =\Lambda_{0}\exp[\mathrm{C´}/(T-T_{2})])$ of electrolyte mixtures.

**Sample**	**EM-0**	**EM-1**	**EM-2**	**EM-3**	**EM-4**	**EM-5**	**EM-6**
*κ*, 273.15 K, [mS cm^-1^]	7.1	1.4	2.0	1.8	1.4	0.1	1.4
*κ*, 293.15 K. [mS cm^-1^]	10.7	2.9	4.0	3.5	2.8	0.4	2.5
*Λ*, 293.15 K. [S cm^2^ mol^-1^]	10.6	2.4	3.5	3.3	2.7	0.4	2.6
*κ*, 313.15 K. [mS cm^-1^]	15.0	5.1	6.8	5.9	4.8	0.9	3.8
*Λ*, 313.15 K. [S cm^2^ mol^-1^]	15.2	4.3	6.0	5.6	4.7	0.9	4.1
*κ*, 333.15 K. [mS cm^-1^]	19.5	7.5	9.7	8.6	7.2	1.5	5.2
*Λ*, 333.15 K. [S cm^2^ mol^-1^]	20.1	6.5	8.8	8.3	7.1	1.6	5.8
*κ*, 353.15 K. [mS cm^-1^]	24.1	10.1	12.9	11.4	9.7	2.2	6.7
*Λ*, 353.15 K. [S cm^2^ mol^-1^]	25.3	9.0	11.9	11.1	9.7	2.3	7.6
*κ*_0_, [S cm^2^ mol^-1^]	203.7	100.4	101.2	106.5	102.2	22.0	56.0
*C*, [K]	-467.9	-393.3	-345.4	-391.6	-413.2	-335.0	-401.6
*Τ*_1_, [K]	133.9	181.6	185.7	177.7	177.8	207.8	164.3
Cor. R^2^. a	1.000	1.000	1.000	1.000	1.000	1.000	1.000
*E*_A,κ_, [kJ mol^-1^] b	12.2	19.8	18.8	18.6	19.6	27.8	15.6
*Λ*_0_, [S cm^2^ mol^-1^]	217.3	88.1	106.0	105.0	105.2	24.8	67.7
*C´*, [K]	-448.7	-377.4	-368.7	-381.0	-406.4	-345.3	-401.9
*Τ*_2_, [K]	144.4	187.9	184.6	183.3	182.4	208.0	168.2
Cor. R^2^. c	1.000	1.000	1.000	1.000	1.000	1.000	1.000
*E*_A,Λ_, [kJ mol^-1^] b	12.5	18.7	17.4	17.6	18.5	24.2	15.2

a Correlation coefficient for fitting of the temperature-dependent specific conductivity data using VFT equation.

b The activation energy values *E*_A, κ (Λ)_ were evaluated using the slope (*-E*_A, κ (Λ)_*/R*) of the Arrhenius plots.

c Correlation coefficient for fitting of the temperature-dependent molar conductivity data.

### 1.5 Anodic aluminum dissolution

See Figs. 1 and 2.

**Fig. 1:**
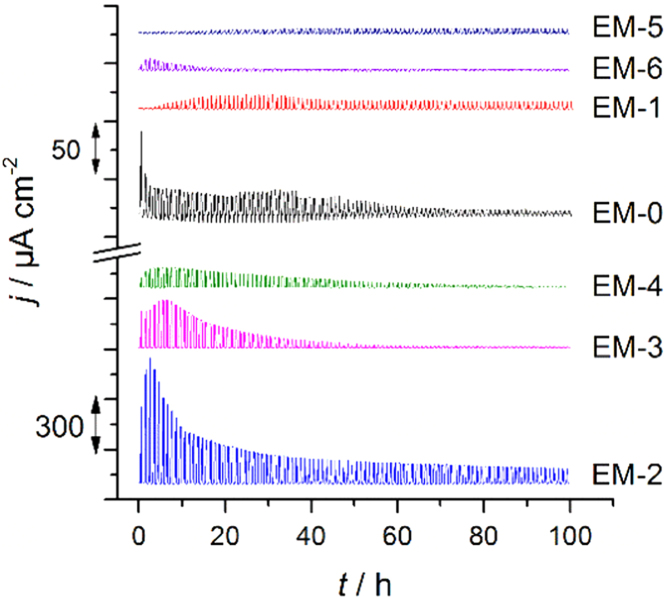
The current density diagram independence of the time for the aluminum dissolution measurements with mixtures EM-*n* (*n* = 0–6). The potential is shifted between 2.5 and 4.3 V vs. Li/Li^+^ with the scan speed 1 mV s^−1^ (corresponding to Fig. 4, Ref. [1]).

**Fig. 2:**
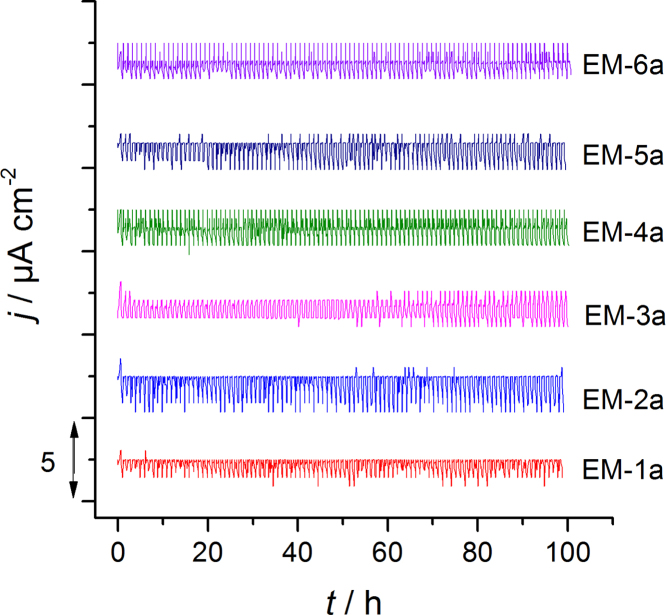
The current density diagram independence of the time for the aluminum dissolution measurements with mixtures EM-*n* (*n* = 1a–6a) with additive LiBOB. The potential is shifted between 2.5 and 4.3 V vs. Li/Li^+^ with the scan speed 1 mV s^−1^ (corresponding to Fig. 5, Ref. [1]).

### 1.6 Cell tests of EM-4 including additives

See Figs. 3 and 4.

**Fig. 3:**
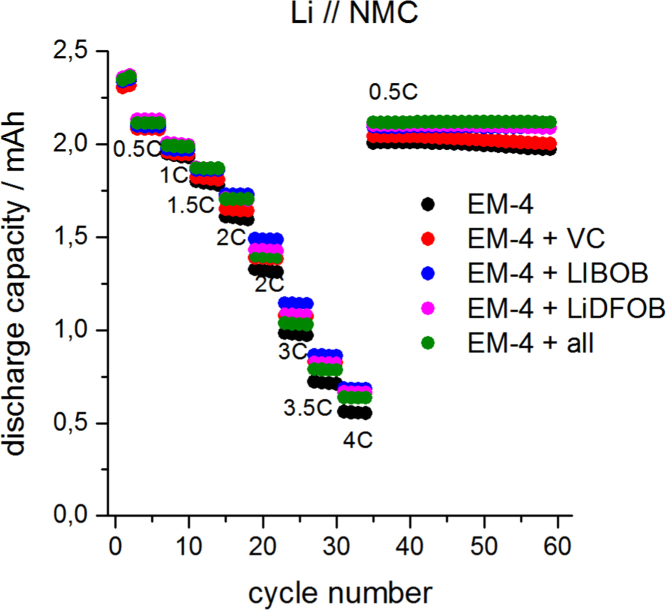
Half cell tests of Li//NMC cells at room temperature (25 °C) in coin cell configuration at various current rates. Four individual cells were averaged.

**Fig. 4:**
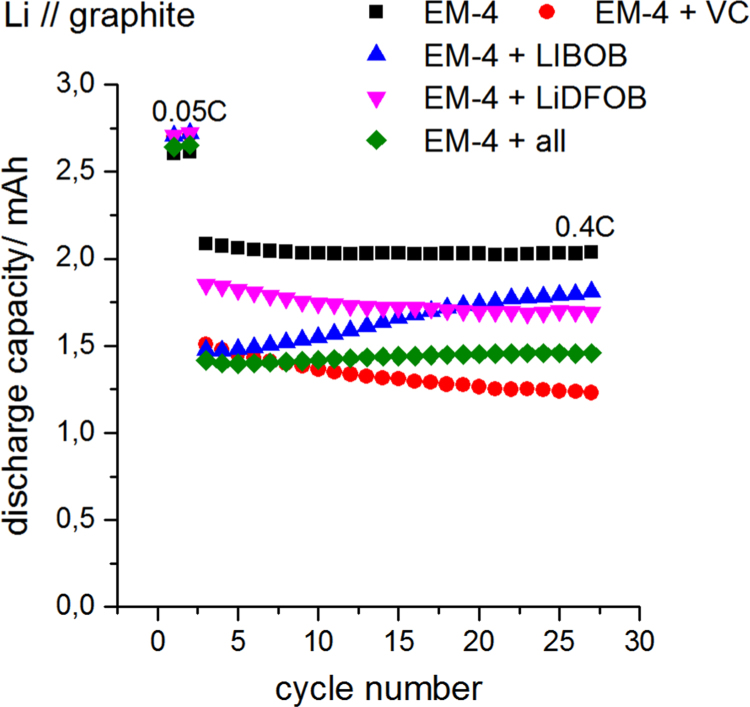
Half cell tests of Li//graphite cells at room temperature (25 °C) in coin cell configuration at various current rates. Four individual cells were averaged.

### 1.7 SEM images of anode sheets after cell cycling

See Fig. 5.

**Fig. 5:**
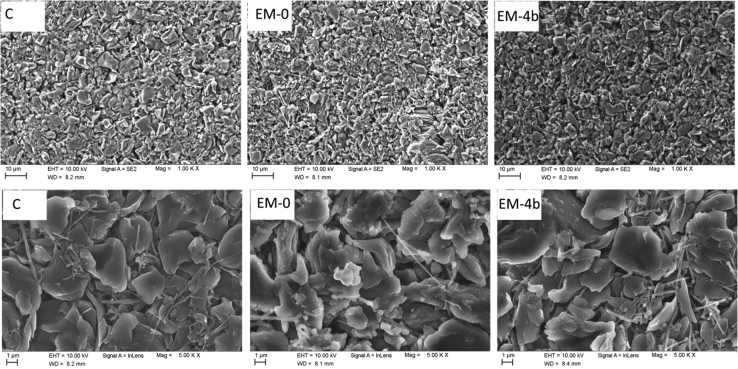
SEM images of the anodes before (C) and after cycling (200 cycles) with electrolyte EM-0 and EM-4b in different magnification (1.000× and 5.000×).

### 1.8 EDX data of the cell cycling

See Fig. 6.

**Fig. 6:**
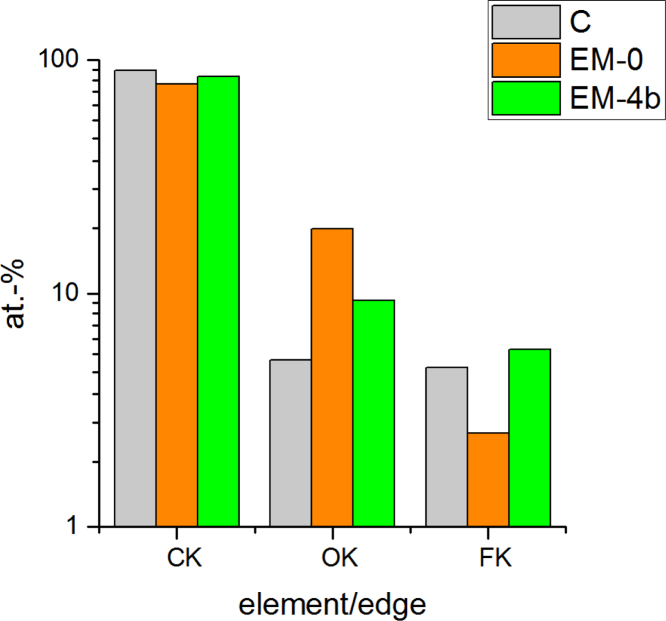
**:** Composition of the surface of the anode before (C) and after (EM-0/EM-4b) cell cycling (200 cycles at 25 °C). The anode sheets were washed with dimethyl carbonate several times before analysis. It was found that Al and Si is present in different amounts due to the separator Separion. These amounts were subtracted out for a better comparison between the cells. Additionally, the amount of conducting salt was subtracted as well based on P (EM-0, LiPF_6_) and S (EM-4b, LiTFSI). Thereafter, the atom-% were recalibrated to 100%.

## 2 Experimental design, materials, and methods

All chemicals and abbreviations are listed in Table 6 and all mixtures are listed in Table 7.

**Table 6:** Chemicals and quality.

Chemical	Supplier	Abbreviation	Purity	
Lithium bis(trifluoromethanesul-fonyl)imide	Sigma-Aldrich	LiTFSI	>99.95%	dried under vacuum at 120 °C for 5 days
lithium bis(oxalato)borate	Sigma-Aldrich	LiBOB		dried under vacuum at 120 °C for 5 days
lithium tetrafluoroborate	ABCR	LiBF_4_	99.997%	used as received
lithium difluoro(oxalato)borate	Sigma-Aldrich	LiDFOB	battery manu-facturing quality	used as received
Ethylene carbonate	Huntsman	EC	ultrapure	used as received
propylene carbonate	ACROS	PC	anhydrous 99.5%	used as received
1,2-butylene carbonate	TCI Europe	1,2-BC	>98.0%	dried at 120 °C by pressing dry air through the solvent
Dibenzyl carbonate	ABCR	DBC	98%	used as received
dipropyl carbonate	BOC Sciences	DPrC	98.9%	dried with 3 Å molecular sieve and clarified by filters
fluoroethylene carbonate	TCI europe	FEC	>98.0%	dried with 3 Å molecular sieve and clarified by filters
vinylene carbonate	Aldrich	VC	97%	dried with 3 Å molecular sieve and clarified by filters
Lithium foils	Alfa Aesar		0.75 mm thick	used as received
Aluminum foils	Hohsen Corp. Japan

**Table 7:** Composition of electrolyte mixtures EM-*n* (*n* = 0–6).

Sample	EM-0	EM-1	EM-2	EM-3	EM-4	EM-5	EM-6
solvents (molar-ratio 1:1)	EC	FEC	FEC	FEC	FEC	FEC	FEC
	DMC		EC	PC	1,2-BC	DBC	DPrC
conducting salt	LiPF_6_	LiTFSI	LiTFSI	LiTFSI	LiTFSI	LiTFSI	LiTFSI
c (conducting salt) [mol kg^-1^]	0.771	0.75	0.75	0.75	0.75	0.75	0.75

The preparation of the electrolytes was performed in an argon-filled glove box (MBraun GmbH) with oxygen and water levels below 0.5 ppm. The reference electrolyte EM-0 (ethylene carbonate, dimethyl carbonate, LiPF_6_, Sigma-Aldrich, battery grade) was used as received.

The conductivity values were measured with a device from RHD instruments and the measurements were done in a humidity controlled chamber (SH-261, ThermoTec Espec; 0–80 °C) by the standard complex impedance method (Zahner Zennium IM6 electrochemical workstation, Kronach, Germany; frequency range: 1 kHz to 1 MHz; ac-offset: 10 mV; the cell constant C was received by measuring a standard solution of 1.413 mS cm^-1^ at 25 °C, Hanna Instruments, HI 70031; *u*(*C*) = 0.01 C).

The dynamic viscosity values were determined using a rotational rheometer (cone/plate geometry, 40/1°, gap of 30 mm, Malvern Gemini HR Nano, Worcestershire, UK) in range of *T* = (15–80) °C (shear rate of 100 s^-1^).

The density values were obtained by measuring electrolyte mixtures (ca. 1 ml) between 20 °C and 80 °C with the device DMA 4500 M from Anton Paar.

To investigate the anodic aluminum dissolution, Swagelok-cells were used with an aluminum foil (*Ø* = 12 mm) as working electrode, lithium foil (*Ø* = 12 mm) as reference electrode and a glass fiber separator (GF/A, *Ø* = 13 mm; electrolyte volume: 40 µl) in between. The cells were assembled inside a glove box and were cycled at the range of 2.5–4.3 V (1 mV s^-1^) at 22–25 °C.

The EDX data were received after subtracting the conducting salt amount based on P (EM0) or C (EM4) and recalibrating to 100%. This assumed that the conducting salt is not constituent of the SEI layer. It must be noted that this is an estimation only to illustrate the F content on the surface arising from solvents and additives.
